# Mapping the needs of healthcare workers caring for COVID-19 patients using the socio-ecological framework: a rapid scoping review

**DOI:** 10.1186/s12960-024-00919-8

**Published:** 2024-05-21

**Authors:** Pinelopi Konstantinou, Vaso Theofanous, Maria Karekla, Angelos P. Kassianos

**Affiliations:** 1 Department of Psychology, University of Cyprus, Nicosia, Cyprus; 2 Department of Nursing, Cyprus University of Technology, 3041 Limassol, Cyprus; 3 Department of Applied Health Research, UCL, London, United Kingdom

**Keywords:** COVID-19, Healthcare workers, Needs, Challenges, Psychological interventions, Socio-ecological models

## Abstract

Undoubtedly, the mental health of healthcare workers (HCWs) was negatively affected because of caring for patients during the COVID-19 pandemic. However, literature is limited on mapping the challenges and needs of HCWs during COVID-19 pandemic. A widely used framework in public health for mapping evidence includes the socio-ecological models, suggesting behavior can be influenced by individual, interpersonal, organizational, and community factors. The aim of this rapid scoping review was to use the socio-ecological model to map and compile lessons learnt from the literature regarding primarily the challenges and needs and secondly available psychological interventions for HCWs caring for COVID-19 patients. PubMed, CINAHL and Scopus databases were searched, with 21 studies finally included examining challenges and needs of HCWs and 18 studies presenting psychological interventions. Organizational-level challenges and needs such as inadequate staff preparation and supplies of protective equipment, flexible work policies and paid rest periods were the most reported. Individual-level challenges and needs included COVID-19-related fears and reduced mental health, whereas interpersonal-related needs included support provision. Community-level challenges included societal stigma. Certain psychological interventions were found to be promising for HCWs, but these were utilized to address only individual-level challenges and needs. Given that well-being entails an interaction of factors, multi-level interventions addressing multiple socio-ecological levels (interpersonal, organizational, community) and that place HCWs in their social context should be administrated to increase and maintain intervention’ effects long-term and possibly aid in better coping with future pandemics.

## Introduction

On March 11, 2020, coronavirus disease (COVID-19) was declared by the World Health Organization [1] as a global pandemic and posed an extremely high risk, burden and negative impact on the physical and mental health especially of those frontline healthcare workers (HCWs) [1–4]. Healthcare systems in many countries at the time of the COVID-19 pandemic were on a brink of collapse, with HCWs exposed to unprecedented psychological strain [5] and experiencing an increased risk for infection and adverse physical health outcomes [3, 4, 6]. Work overload, social isolation, fear of infecting friends and family, physical exhaustion and the constant need for taking ethically difficult decisions were among the factors contributing to deteriorating mental health [2, 4, 5, 7]. HCWs were particularly at risk of experiencing increased symptoms of burnout, anxiety, depression, post-traumatic stress and insomnia [6, 8–13]. HCWs had to face several challenges during the COVID-19 pandemic including high workload, death of colleagues and patients, and being stigmatized by community members, whereas they reported needs for adequate rest, appreciation from management, and psychological support [3, 5, 9, 14, 15]. Therefore, their challenges and needs are multi-factorial influenced for example by work-related conditions (e.g., excessive workload) and individual-based cognitions and feelings (e.g., reduced mental health).

One of the earliest ecological models in psychology and public health is the Bioecological Model of Human Development [16]. According to the model [16, 17], an individual’s development is influenced by the microsystem (interactions with immediate environment like family), mesosystem (connections between different microsystems such as work and family), exosystem (indirect influence by an individual’s environment), macrosystem (cultural context of the individual) and chronosystem (changes in individual and environment across time). Socio-ecological models [18–21] such as the “Rainbow Model” [22, 23] are widely used in public health for mapping evidence (e.g., factors affecting mental health). They suggest that an individual’s behavior, emotions and relationships can be influenced by: (a) individual (i.e., choices, beliefs, attitudes, demographic characteristics), (b) interpersonal (i.e., formal or informal support systems such as family and friends), (c) organizational (i.e., organizational settings that exist outside home such as workplace), and (d) community factors (i.e., social interaction, political and psychological). It is of crucial importance to map the evidence from the literature on challenges and needs of HCWs caring for COVID-19 patients based on well-established socio-ecological models so as to possibly contribute to translating into policymaking actions and interventions.

A range of psychological interventions available to HCWs during COVID-19 were examined in previous reviews. They found that mindfulness training [14, 24], problem solving [24], Cognitive Behavioral Therapy (CBT) [6, 24], and Acceptance and Commitment Therapy (ACT) [6] were effective on improving mental health symptoms such as anxiety, depression, and post-traumatic stress. However, in order to maximize the effects of an intervention, the needs of HCWs at multiple levels (e.g., including contextual) should be addressed. The importance of conducting multi-level research was further supported by the guidelines proposed by the Task Force of the Association of Contextual Behavioral Science (ACBS) [25], which suggested that research should be more experimental, multi-level, process-based, and multi-dimensional.

Currently, there is an absence of reviews mapping evidence on the challenges and needs of HCWs caring for COVID-19 patients to different socio-ecological levels (e.g., individual, interpersonal, organizational, community). The aim of this rapid scoping review is to map and compile lessons learnt from the literature regarding the challenges and needs of HCWs caring for COVID-19 patients during the pandemic based on socio-ecological models. A secondary aim of this review is to investigate what type of psychological interventions were utilized and are effective for HCWs during the COVID-19 pandemic.

## Method

The review followed the PRISMA guidelines for reporting scoping reviews [26]. The protocol of this study and the data supporting the findings are available in Open Science Framework (OSF; DOI: 10.17605/OSF.IO/5KBHD).

### Eligibility criteria

Published and unpublished (e.g., dissertations) peer-reviewed studies were eligible for selection. The PICO method was used to determine the inclusion criteria for this review [27]: (a) P (Participants): Working as an HCW during COVID-19 that according to the World Health Organization [28] includes general medical practitioners, nursing professionals, psychologists, physicians, and physiotherapists. Students of any of these specialties and medical residents were also eligible; (b) I (Intervention): Report any psychological intervention available for or examine the challenges and needs of HCWs; (c) C (Comparison): Only studies examining psychological interventions had to compare an intervention group with control or, if no control group was used, the study should have utilized a design with pre–post intervention comparisons or examined the feasibility and acceptability of the intervention; and (d) O (Outcome): Examine either the challenges and needs or psychological interventions for HCWs caring for COVID-19 patients. Additionally, included studies examining the challenges and needs of HCWs had to utilize either qualitative (i.e., interview, focus groups) or quantitative (i.e., randomized controlled trial (RCT), correlational, and experimental) design. Challenges were defined as the problems experienced requiring great mental or physical effort in order to be done successfully during the COVID-19 pandemic whereas needs were defined as the conditions required for improved health and quality of life [29].

Studies were excluded if they were: (a) published in language other than English; (b) reviews, editorials, conference abstracts, or case studies; and (c) published before 2020 when COVID-19 was declared a pandemic.

### Search strategy

Relevant studies published during the period of COVID-19 pandemic (2020–2024) were identified by searching the databases of PubMed, CINAHL and Scopus. Searches were conducted until end of March 2024. Existing relevant meta-analyses and reviews were also examined for additional eligible studies. A defined search strategy was undertaken using the following terms based on title and abstract: “COVID-19” or “COVID 19” or “SARS-COV-2” or “coronavirus” combined with the terms “healthcare professionals”, or “healthcare providers”, or “doctors”, or “nurses”, or “healthcare workers”, or “physicians”, and “need” or “challenge” or “intervention” or “treatment”. The full search strategy is available as Appendix.

### Inter-rater reliability (IRR)

Articles were screened for eligibility at all screening stages by the first author. At all stages, an additional author (VT) screened 20% of the studies, independently. Inter-rater reliability (IRR) was calculated using the percent agreement and Cohen’s kappa [30]. An almost perfect agreement was observed between the two screeners in title-abstract (*IRR* = 90%; *k* = 0.95) and substantial agreement in full-text screening (*IRR* = 69%; *k* = 0.80). Any discrepancies were resolved in research team consensus meetings.

### Data extraction and synthesis

A data charting form was used to extract the data. From all included studies, a mixture of general information about the characteristics of the study and population and specific information relating to the aims of this scoping review were extracted. A narrative synthesis approach [31, 32] was used to describe, analyze, summarize and interpret included study findings. Since we included both quantitative and qualitative studies, a mixed methods framework was used to synthesize the data, which is a convergent synthesis design where both types of data are collected and analyzed simultaneously [31]. Based on the data type provided by each study, the results-based convergent synthesis design was used in which both data types were analyzed and presented separately and then collated together. The themes reported by qualitative studies were extracted, whereas statistical data were extracted from the quantitative studies. The socio-ecological model was used to summarize and cluster the challenges and needs of HCWs into individual, organizational, interpersonal and community factors [18, 19].

## Results

### Study characteristics

**Fig. 1:**
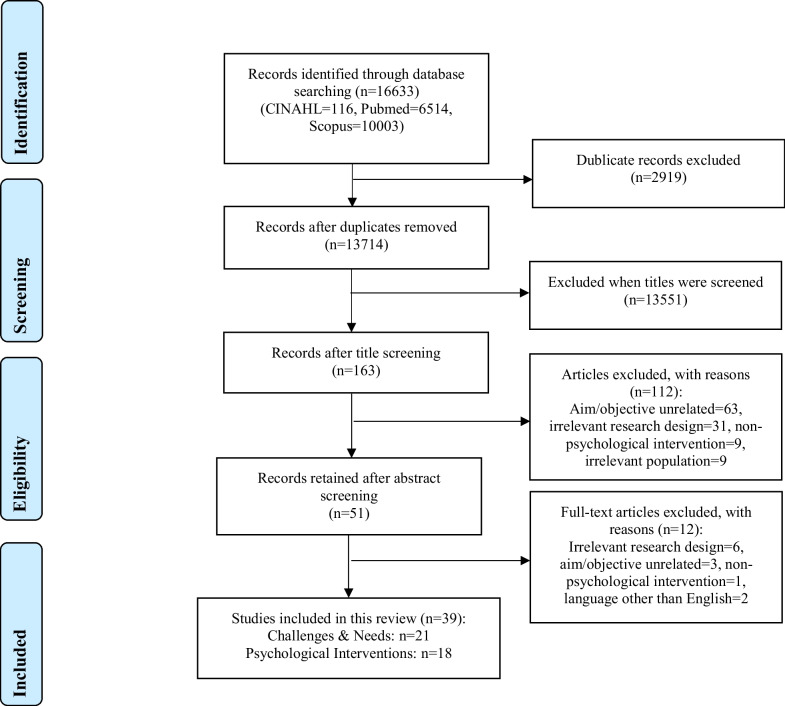
Flow diagram of information detailing the database searches, the number of titles and abstracts screened and excluded, and the full texts retrieved and excluded

A total of 16,633 studies were identified in initial search. After removing duplicates and screening the titles, 51 studies were screened for full text and 21 were included to examine the challenges and needs of HCWs, whereas 18 examined psychological interventions available for HCWs (see Fig. 1 for a detailed flow diagram including reasons for exclusion). The characteristics of the included studies examining the challenges and needs are presented in Table 1, whereas those examining psychological interventions are shown in Table 2.

**Table 1:** Characteristics of included studies for challenges and needs (N = 21)

Study	Country	Aim	Research design	Sample size	Age (M, SD)^a^	Gender (n, % females)	Specialty of HCWs
Abba et al. [70]	Nigeria	To explore the experiences of HCWs on managing hospitalized patients with COVID-19	Qualitative with interviews	2021	35.6 (15.4)	4 (36.4)	Nursing professionals: 5 (45.5%)
Ali & Kumar [33]	India	To assess issues, challenges and coping strategies of HCWs	Cross-sectional	759	25–44: 77.0%	384 (51.0)	Nursing professionals: 325 (43.0%) Doctors in a COVID-19 ward: 270 (36.0%)
Banerjee et al. [38]	India	To explore the experiences of frontline physicians	Qualitative with interviews	172	29.2 (3.8)	62 (36.0)	General physicians: 74 (43.0%)
Creese et al. [37]	Ireland	To explore changes on well-being of doctors and to provide recommendation on its better support	Qualitative with interviews	48	NR	37 (77.0)	Specialist Internal Medicine: 12 (32.0%) Emergency and General Internal Medicine: 11 (23.0%)
Cumberland et al. [35]	USA	To assist one US healthcare system with the implementation of needs assessment among frontline healthcare workers	Qualitative with focus groups	357	NR	NR	Nursing professionals: 241 (68.0%)
Das Pooja et al. [40]	Bangladesh	To describe the challenges faced by frontline HCWs, and what motivated them to continue providing service	Qualitative with interviews and focus groups	18	NR	9 (50.0)	Physicians: 11 (61.1%)
Dempsey et al. [41]	Ireland	To explore the experiences of undergraduate nursing students who worked clinically during COVID-19 pandemic	Qualitative with focus groups	47	18–28: 83.0%	46 (97.9)	Nursing students (100%)
Gursoy et al. [42]	Turkey	To examine the experiences and perceptions of nurses who provided care for patients with COVID-19	Qualitative with interviews	11	28.9 (NR)	NR	Nursing professionals (100%)
Hameed et al. [39]	Pakistan	To explore the mental health impact and needs of public sector HCWs	Qualitative with interviews	56	46.6 (10.6)	9 (16.1)	Hospital managers: 40 (71.4%) Health service providers (e.g., nurses): 16 (28.5%)
Jimu et al. [66]	Africa	To explore the lived experiences of frontline HCPs during the peak of the second wave of COVID-19	Qualitative with interviews	15	32.5 (NR)	15 (100%)	Nursing professionals (100%)
Konduru et al. [44]	India	To examine the experiences of HCWs during COVID-19	Qualitative with interviews	5	45.2 (NR)	3 (60.0)	Doctors: 2 (40.0%) Nursing professionals: 1 (20.0%) Paramedic: 1 (20.0%) Midwife: 1 (20.0%)
Lee et al. [65]	South Korea	To explore the experiences of frontline nurses combating COVID-19	Qualitative with interviews	14	38.3 (NR)	11 (78.6)	Nursing professionals: 9 (64.3%)
Liu et al. [68]	China	To explore the experiences of HCWs who provided care to COVID-19 patients	Qualitative with interviews	15	27.83 (5.43)	10 (66.7)	Nursing professionals (100%)
Moyo et al. [64]	Africa	To understand the needs faced by HCWs during COVID-19	Qualitative with interviews	10	31–35: 60.0%	8 (80.0)	Nursing professionals: 6 (60.0%)
Mukhaimer et al. [67]	Bahrain	To explore the lived experiences of nurses during COVID-19	Cross-sectional (online survey)	627	34.88 (7.65)	495 (78.9)	Nursing professionals: 542 (86.4%)
Nyandeni et al. [45]	Africa	To explore the lived experiences of frontline HCPs during the peak of the second wave of COVID-19	Qualitative with interviews	13	24–55: 100%	12 (92.0)	Nursing professionals (100%)
Ralph et al. [43]	Canada and USA	To capture the recommendations of nursing professionals providing frontline care	Qualitative with interviews	36	NR	31 (86.0)	Nursing professionals (100%)
Rodríguez-Almagro et al. [71]	Spain	To describe the perceptions and experiences of nursing students	Qualitative with interviews	20	25.4 (NR)	12 (60.0)	Nursing students (100%)
Romate & Rajkumar [36]	India	To understand the experiences, challenges, psychological well-being and needs of HCWs	Qualitative with interviews	221	32.52 (21.9)	92 (41.6)	Nursing professionals: 100 (45.2%)
Setiawan et al. [69]	Indonesia	To explore strategies for overcoming challenges in caring for COVID-19 patients	Qualitative with interviews	28	35.0 (4.0)	15 (53.6)	Nursing professionals: 16 (57.1%)
Siddiqui et al. [34]	UK	To identify causes of anxiety in HCWs, to assess the well-being support and to identify their unmet support needs	Cross-sectional (online survey)	558	25–65: 94.0%	430 (77.0)	General physicians: 184 (33.0%)

HCW = healthcare worker; NR = not reported

^a^For studies not reporting mean age, frequencies with the highest percentage are presented instead

**Table 2:** Findings on the psychological interventions used for HCWs (n = 18)

Study	Country	Research Design	Sample Size	Specialty of HCWs	Location that HCWs lived/worked	Intervention Information				Findings
						Format	Length (Weeks)	Intervention Group	Control Group	
Al Ozairi et al. [51]	UK	Quasi-experimental study	56	Physicians (100%)	Kuwait	Group	2	MBI	No CG	• Sign. improvements in mindfulness, anxiety and depression
AlQarni et al. [55]	Saudi Arabia	RCT	125	Nurses: 75 (60.0%)	Eastern province, Saudi Arabia	Online individual	2	MBI	PMR	• IG sign. greater improvement in psychological well-being than CG • Both groups sign. Equal reduction on state anxiety
Bureau et al. [62]	France	Qualitative with interviews	10	Nursing professionals: 3 (30.0%)	Alsace region	Website	1	My Health too: mindfulness, acceptance, values, self-compassion	No CG	• Website was easy to use and understand, useful in inducing calm and in practicing self-compassion
Fiol-DeRoque et al. [60]	Spain	RCT	482	Nursing professionals: 161 (33.4%) Physicians: 153 (31.7%) Nurse assistants: 147 (30.5%)	Spain	Mobile app	2	PsyCovidApp: based on mindfulness and CBT	Control app: recommendations about mental health care	• No sign. differences between groups at post-treatment • Sign. differences between groups only on HCWs receiving psychotherapy or psychotropic medications on reducing post-traumatic stress, insomnia, anxiety, and stress at post-treatment
Gnanapragasam et al. [59]	UK	RCT	894	Nursing professionals: 210 (23.5%) Administrative and clerical staff: 175 (19.6%) Healthcare/nursing assistant: 80 (9.0%)	UK	Mobile app	8	Foundations app: based on CBT, mindfulness, relaxation and positive psychology	Waitlist	• IG sign. reduction in psychiatric morbidity symptoms and insomnia and improvement in well-being
Gupta et al. [54]	India	RCT	35	Interns/postgraduate trainee frontline HCWs: 9 (63.2%)	Central India	Individual	1–1.5	Tele-counseling eclectic psychotherapy	General education	• No group sign. group differences on depression, anxiety and stress • Sign. improvement over time on depression, anxiety and stress
Han et al. [50]	China	Quasi-experimental study	226	Nursing professionals (100%)	Xuzhou	Group	10	ACT	No CG	• Sign. improvements in mental health symptoms • No sign. results for stress and psychological resilience
Hosseinzadeh Asl [52]	Turkey	RCT	49	Social workers (100%)	Ankara	Individual	4	Mindfulness exercises	Waitlist	• IG sign. higher psychological flexibility and self-compassion and lower depression vs. CG at post-treatment and follow-up • No sign. group differences in anxiety and stress
Keng et al. [58]	Singapore	RCT	80	Nursing professionals: 47 (58.8%)	Singapore	Mobile app	3	Mindfulness exercises	Active CG (playing cognitive games)	• No sign. between-group changes from pre- to post-treatment • From pre- to 1-month follow-up, sign. greater improvements in IG on fear of COVID-19, compassion, mindfulness, and forward digit span task
Li et al. [63]	China	RCT	270	NR	Guangzhou	Website	4	SH + : Self-managed stress management program based on ACT	Waitlist	• IG sign. lower stress at 3-month follow-up than CG • IG sign. reduced stress over time • IG sign. improved depression, insomnia, positive affect and self-kindness than CG
Miyoshi et al. [53]	Japan	Experimental study	18	Nursing professionals: 7 (53.8%)	Okayama	Individual	12	Yoga and mindfulness	No CG	• No sign. changes for general health, burnout, resilience, self-compassion, empathy
Morina et al. [56]	Switzerland	RCT	160	Physicians: 64 (41.3) Nurses: 61 (38.1)	Zurich	Online individual	4	RECHARGE: Problem solving strategies, strategies for restrictions of social distancing, relapse prevention	ATAU: Reference on 2 websites for coping strategies of distress	• IG sign. greater reduction in psychological distress, worry, burnout, and moral injury distress than CG • No sign. results at 6 months follow-up
Mosazadeh et al. [49]	Iran	RCT	30	Nursing professionals (100%)	Tehran	Group	8	ACT	No intervention	• IG sign. lower occupational stress and anxiety vs. CG at post-treatment
Otared et al. [48]	Iran	RCT	40	Healthcare workers (100%)	Tabriz	Group	8	ACT	Waitlist	• IG sign. lower depression and anxiety and higher quality of life vs. CG at post-treatment
Rizzi et al. [57]	Italy	RCT	225	Nurse: 92 (41.0%) Doctor: 90 (40.0%)	Pavia	in-person or online individual	NR	Brief DBT	No intervention	• Sign. decrease in PTSD symptoms in all groups across time • No sign. difference between CG and IGs on PTSD symptoms • IG sign. greater reductions in PTSD symptoms than CG, but only in participants with severe PTSD symptoms
Rodriguez-Vega et al. [47]	Spain	Exploratory study	149	Nursing professionals: 52 (46.0%)	Madrid	Group	1 day	Mindfulness exercises	No CG	• Participants perceived intervention as being helpful for reducing stress
Trottier et al. [61]	Canada	Uncontrolled trial	21	Nursing professionals: 11 (52.4%)	Ontario	Online platform	8	RESTORE: online intervention based on CBT	No CG	• At post-treatment, sign. improvements in anxiety, depression and PTSD severity • Intervention was reported as feasible and safe
Tuna & Ermis [46]	Turkey	RCT	58	Nursing professionals: 29 (50.0%)	Istanbul	Group	8	Mental health support program based on CBT	TAU	• IG sign. lower anxiety and depression at post-treatment vs. CG • No sign. differences in insomnia

CBT = cognitive behavioral therapy; CG = control group; HCW = healthcare worker; IG = intervention group; PMR = progressive muscle relaxation; RESTORE = recovering from extreme stressors through online resources and E-health; TAU = treatment as usual

**Fig. 2:**
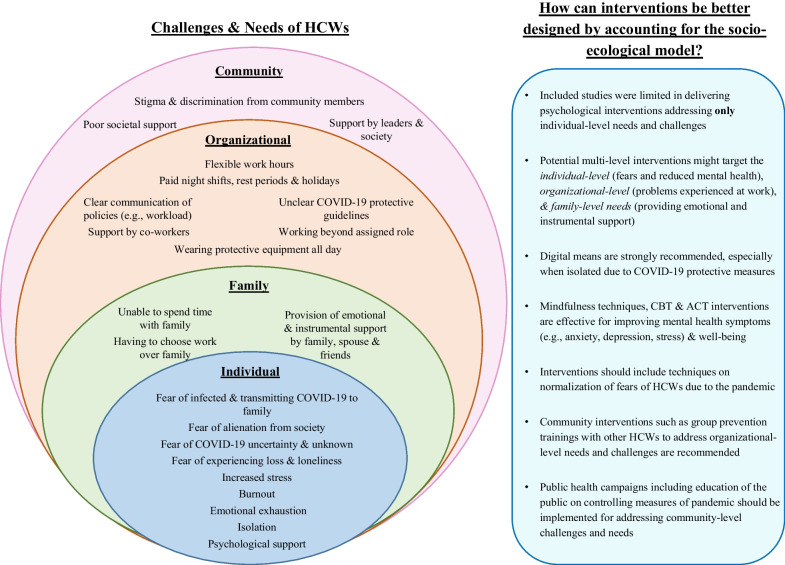
Social-ecological framework of challenges and needs of HCWs caring for COVID-19 patients and potential psychological interventions to address them

Studies were published between 2020 and 2024 and conducted in a range of countries. Specifically, studies examining challenges and needs of HCWs were conducted mainly in India (*n* = 4, 19.0%), USA (*n* = 2, 9.5%), Ireland (*n* = 2, 9.5%) and Africa (*n* = 2, 9.5%), whereas those examining psychological interventions were conducted in Iran (*n* = 2, 11.1%), Spain (*n* = 2, 11.1%), Turkey (*n* = 2, 11.1%), China (*n* = 2, 11.1%) and UK (*n* = 2, 11.1%). Studies examining challenges and needs implemented mostly a qualitative design utilizing interviews or focus groups (*n* = 18, 85.7%) or were cross-sectional studies utilizing quantitative methods (*n* = 3, 14.3%). In contrast, all studies examining psychological interventions implemented a quantitative design utilizing mostly a clinical trial (*n* = 12, 66.7%). Overall, in most studies the sample was comprised mainly by HCWs specialized in nursing (*n* = 26, 66.7%) or general physicians (*n* = 5, 12.8%). The challenges and needs were mapped into four ecological levels: individual, organizational, interpersonal, and community (see Table 3 for each study and Fig. 2 for a summary).

**Table 3:** Findings on the challenges and needs of included studies based on socio-ecological models (n = 21)

Study	Country	Challenges and needs			
		Individual	Interpersonal	Organizational	Community
Abba et al. [70]	Nigeria	–	–	Challenges: • Some patients did not cooperate well • Lack of protective equipment • Inadequate feeding and accommodation	Challenges: • Social stigma and isolation
Ali & Kumar [33]	India	Most-reported challenges: • 69% afraid of contracting COVID-19 • 52% felt emotionally tired • 50% fear of alienation from society	–	Most-reported challenges: • 74% unclear COVID-19-related guidelines • 80% wearing protective equipment every day • 51% lack of incentives provided to them	Most-reported challenges: • 49% issues such as poor support from society • 41% stigma and discrimination from society
Banerjee et al. [38]	India	Challenges: • Fear of infection and uncertainty • Sense of guilt • Loneliness and burnout • Social isolation	–	Needs: • Flexible work policies • Administrative measures for better medical protection • Effective risk communication for health	Challenges: • Stigma from society Needs: • Social inclusion
Creese et al. [37]	Ireland	Challenges: • Decline in mental well-being due to anxiety, emotional exhaustion, guilt, and isolation	–	–	–
Cumberland et al. [35]	USA	Challenges: • Fear of the unknown associated with pandemic anxiety, stress, exhaustion and depression	–	–	–
Das Pooja et al. [40]	Bangladesh	Challenges: • Fear of transmitting COVID-19 to family • Authenticity and/or quality of COVID-19 information • Interaction with patients and their families	Challenges: • Unable to spend time with family • Choosing work over family	–	Challenges: • Stigma from society
Dempsey et al. [41]	Ireland	Challenges: • Fear of infection and transmitting to family	–	Challenges: • Insufficient/Not adequate staffing • Communication problems with patients due to wearing protective equipment	–
Gursoy et al. [42]	Turkey	Challenges: • Physical and psychological fatigue • Fear due to uncertainty of COVID-19 • Fear of death due to increasing spread of the disease Needs: • Psychological support	–	Challenges: • Longer working hours • Physical needs (e.g., difficulty breathing while on a mask) • Increased work load • Lack of management support Needs: • Financial support • Improvements in working conditions	Challenges: • Social stigma and isolation
Hameed et al. [39]	Pakistan	Challenges: • Fear of infection • Fear of transmitting COVID-19 to family • Social isolation • Anxiety due to uncertainty of COVID-19 • Stress Needs: • Psychological support	–	Needs: • Safe working conditions • Paid rest periods • Appreciation and motivation to work	Challenges: • Stigma from society
Jimu et al. [66]	Africa	Challenges: • Fear of infection and transmitting to family • Experience of loss and feelings of helplessness	–	Challenges: • Wearing protective equipment every day • Scarcity of resources	Challenges: • Social stigma
Konduru et al. [44]	India	Challenges: • Fear of infection and transmitting to family • Fear of being able to treat patients adequately • Feelings of helplessness, hopelessness, anger • Dissatisfaction upon not getting recognition Needs: • Psychological support • Rest	–	Challenges: • Lack of supplies • Insufficient staffing • Lack of peer support • Inferior quality of care Needs: • Increase in workforce • Adequate supply of protective equipment	–
Lee et al. [65]	South Korea	Challenges: • Fear of infection • Stress due to intensity of work • Feelings of hopelessness • Fatigue	Challenges: • Reduced support from family and friends	Challenges: • Communication difficulties with staff and patients • Increased workload • Working beyond the scope of assigned role • Insufficient support or reward • Physical depletion because of protective clothing	Challenges: • Social stigma
Liu et al. [68]	China	Challenges: • Fear of infection and transmitting to family • Extreme stress	–	Challenges: • Caring for patients being critically ill and contagious • Wearing protective equipment every day • Insufficient training about infectious epidemics Needs: • Improvement of protective equipment • Quick hospital responses on future epidemics (e.g., cabin hospitals for isolation)	–
Moyo et al. [64]	Africa	Challenges: • Feelings of fear, anxiety and stress	Challenges: • Alienation by family members Needs: • Support from family	Challenges: • Suboptimal staff preparation • Lack of institutional support • Lack of support from colleagues • Inadequate protective equipment and limited medical supplies	Challenges: • Social stigma and discrimination
Mukhaimer et al. [67]	Bahrain	–	–	Challenges: • Physical needs (e.g., difficulty breathing while on a mask) • Limited communication due to protective equipment • Longer working hours	–
Nyandeni et al. [45]	Africa	Challenges: • Fear of infection and transmitting in family • Social isolation Needs: • Psychological support	–	Challenges: • Scarcity of resources • Lack of managerial support Needs: • Managerial and organizational support	Challenges: • Stigma from society
Ralph et al. [43]	Canada and USA	Needs: • Psychological support	–	Needs: • Clear, consistent and transparent communication related to sick leave and workload • Leadership style that embodied visibility, availability and careful planning • More resilient healthcare supply chain • Pay equity	–
Rodríguez-Almagro et al. [71]	Spain	–	–	Needs: • Paid night shifts and holidays • Working conditions and contracts as promised	–
Romate and Rajkumar [36]	India	Challenges: • Fear of infecting family members • Increased psychological distress and burnout • Experience of loss and feelings of helplessness Needs: • Psychological support	Challenges: • Unable to spend time with family • Choosing work over family Needs: • Family and friends as emotional and instrumental support • Spousal support: Active agent in providing emotional support	Challenges: • Scarcity of resources • Working beyond the scope of assigned role • Communication issues with patients and their families Needs: • Support provided by superiors • Support from co-workers: shared experiences • Security, resources, financial and informational support	Challenges: • Stigma from society Needs: • Support from society
Setiawan et al. [69]	Indonesia	Challenges: • Physical and psychological fatigue	–	Challenges: • Difficulties in working with protective equipment • Insufficient training for handling COVID-19 and protective equipment • Difficulties in carrying out health education and assessment towards patients and families • Limited resources (e.g., insufficient staff, protective equipment, wards for COVD-19 patients)	–
Siddiqui et al. [34]	UK	Challenges: • Only 41% felt there was adequate psychological support • Increased anxiety levels	–	Needs: • Effective leadership and peer support	–

HCW = healthcare worker

### Individual-related

Individual-level challenges were reported by 18 studies conducted in India (*n* = 4, 22.2%), Africa (*n* = 3, 16.6%), Ireland (*n* = 2, 11.0%), USA (*n* = 2, 11.0%), Bangladesh (*n* = 1, 5.6%), China (*n* = 1, 5.6%), Indonesia (*n* = 1, 5.6%), Pakistan (*n* = 1, 5.6%), South Korea (*n* = 1, 5.6%), Turkey (*n* = 1, 5.6%), and UK (*n* = 1, 5.6%). Challenges included mainly fear (78%) and reduced mental health (78%) due to the COVID-19 pandemic [33–42]. Specifically, HCWs faced mostly the fear of contracting COVID-19 and transmitting it to their family members, and the uncertainty that comes with the disease (i.e., they were unaware of the nature and the consequences of the disease due to lack of knowledge about the novel virus), fear of alienation from society, and fear of death due to COVID-19, and experiencing loss and of experiencing loneliness [33, 35, 36, 38–42]. They also reported increased stress, burnout, emotional exhaustion, psychological and physical fatigue, sense of guilt due to the inability to save patients, and isolation due to the COVID-19 protective measures [33, 34, 36–39, 42]. In six studies (33%) conducted in Africa, Pakistan, India, Turkey, and USA, HCWs reported needing psychological support to manage their mental health [36, 39, 42–45]. To be specific, HCWs highlighted the need for counselling services to reduce the stress and anxiety they were experiencing due to the COVID-19 pandemic and to learn coping strategies for dealing with pandemic situations more effectively.

With respect to the psychological interventions being available for HCWs during the COVID-19 pandemic (Table 2), included studies addressed only individual-based challenges and needs, particularly to improve mental health symptoms. In all of the studies (*n* = 18), HCWs worked or lived in primarily urban areas (e.g., Zurich, Pavia, Istanbul, Ontario, Okayama). In the majority of studies (*n* = 15, 83.3%), interventions were administered for a period of two months or less with median duration in weeks being 4.0 (*SD* = 3.5). Interventions were mostly compared to a control group (*n* = 12, 85.7%), such as a waitlist or no-intervention control (*n* = 6 out of 12, 50.0%). In six studies (33.3%), individuals received a group psychological intervention with other HCWs [46–51], whereas in six studies (33.3%), individuals received 1:1 online or telephone counseling from a therapist [52–57]. Interventions were also delivered digitally through developed applications (*n* = , 16.7%) [58–60] including written and audiovisual psychological exercises (e.g., mindfulness, CBT techniques), a developed platform [61] with videos, interactive exercises with written information, a developed website [62] with psychoeducational videos and exercises and a web-based stress management intervention [63] based on ACT as developed by WHO including audiorecordings and illustrated exercises. With respect to the interventions provided, a range of psychological interventions was reported. Most studies delivered CBT (*n* = 4, 22.2%), mindfulness exercises (*n* = 4, 22.2%) and ACT (*n* = 4, 22.2%). A summary of findings of their reported effectiveness can be found in Box 1, whereas for each study in Table 2.

Box 1: Effectiveness of included psychological interventions

- CBT [46, 59–61] and ACT [48–50, 63] resulted in significant improvements in anxiety, depression, insomnia, positive affect, and stress compared to control groups (e.g., waitlist, treatment as usual (TAU)).
- Mindfulness exercises [47, 52, 53, 58] resulted in significantly improving depressive symptoms.
- Tele-counseling eclectic psychotherapy (motivational interviewing, relaxation, life skill and problem-solving training) [52] resulted in reduced depression, anxiety, and stress across time.
- The French website “My Health too” (an online CBT intervention) [62] was reported as feasible, acceptable and useful in inducing relaxation and for practicing self-compassion in HCWs who faced high levels of stress.
- A brief intervention on Dialectical Behavior Therapy (DBT) [57] resulted in reductions in post-traumatic disorder symptoms compared to the no-intervention control group, but only in participants with severe symptomatology.
- The RECHARGE online intervention (strategies on problem solving, relapse prevention in stressful situations) [56] led to greater reduction in distress, worry, and burnout than active treatment-as-usual group at post-treatment.

### Interpersonal-related

Interpersonal-related challenges that HCWs faced were reported by four studies (19.0%) conducted in Africa [64], Bangladesh [40], India [36] and South Korea [65] including alienation by family members, being unable to spend time with family, having to choose work over family and reduced support that HCWs received by family, peers and friends. Interpersonal-related needs of HCWs were reported by two studies (9.5%) including family, spouse and friends providing emotional and instrumental support (e.g., by taking care of children, assisting in household chores), so as to motivate or support HCWs to go to work during COVID-19 [36, 64].

### Organizational-related

Challenges at the organizational-level were reported by 13 studies (61.9%) conducted in Africa (*n* = 3, 23.05%), India (*n* = 3, 23.05%), Bahrain (*n* = 1, 7.7%), China (*n* = 1, 7.7%), Indonesia (*n* = 1, 7.7%), Ireland (*n* = 1, 7.7%), Nigeria (*n* = 1, 7.7%), South Korea (*n* = 1, 7.7%), and Turkey (*n* = 1, 7.7%). Challenges included unclear COVID-19 guidelines at the hospitals regarding protective measures taken, scarcity of protective equipment and limited medical supplies, suboptimal staff preparation for COVID-19, working beyond assigned role (e.g., physicians had to take nursing roles due to shortage of staff and absence of family caregivers), longer working hours, limited communication due to protective equipment, increased workload, lack of managerial support and wearing protective equipment every day for multiple hours [33, 36, 41, 42, 44, 45, 64–70]. Organizational-level needs were reported by 10 studies (47.6%) conducted in India (*n* = 3, 30.0%), Africa (*n* = 1, 10.0%), China (*n* = 1, 10.0%), Pakistan (*n* = 1, 10.0%), Spain (*n* = 1, 10.0%), Turkey (*n* = 1, 10.0%), UK (*n* = 1, 10.0%), and USA (*n* = 1, 10.0%). Specifically, needs reported by HCWs included feeling appreciated at work, support by superiors through listening to their fears and concerns and co-workers such as sharing experiences, flexible working hours, safe and secure working conditions such as administrative measures for better protection from COVID-19, improvement of protective equipment, paid night shifts, rest periods and holidays, and clear communication of policies related to risk, workload and sick leave [34, 36, 38, 39, 42–45, 68, 71].

### Community-related

Community-level challenges were reported by 11 studies (47.5%) conducted in Africa (*n* = 3, 27.25%), India (*n* = 3, 27.25%), Bangladesh (*n* = 1, 9.1%), Nigeria (*n* = 1, 9.1%), Pakistan (*n* = 1, 9.1%), South Korea (*n* = 1, 9.1%), and Turkey (*n* = 1, 9.1%). Challenges included mainly poor societal support (e.g., community members did not recognize HCWs’ contribution during the pandemic) and stigma, isolation and discrimination from society as they had to work in COVID-19 wards, and community members feared that they will contract COVID-19 from HCWs [33, 36, 38–40, 42, 45, 64–66, 70]. Needs were reported by two studies (18.2%) with HCWs reporting support provided by the society by recognizing their contribution in providing support to people and following public protocols for controlling the spread of COVID-19 so as to reduce the burden on them [36, 38].

## Discussion

In this review, 21 studies were included examining the challenges and needs of HCWs caring for COVID-19 patients, and 18 studies examining the psychological interventions available. The socio-ecological models, were used to synthesize the evidence [18, 19, 72]. A range of challenges and needs were identified with HCWs reporting mostly organizational-level factors such as flexible working hours. However, included psychological interventions addressed only individual-based challenges and needs (i.e., mental health symptom improvement), suggesting the importance of developing and administrating multi-level interventions targeting the various factors (interpersonal, organizational, community) influencing well-being [15, 73].

At the individual-level the most reported challenges were fears related to the COVID-19 pandemic such as being infected and transmitting COVID-19 to family members, as well as the uncertainty and mental health symptoms such as increased stress, burnout, fatigue and emotional exhaustion. This is not surprising, as HCWs were experiencing excessive workload, were under immerse pressure and were frequently exposed to infected individuals [3, 4, 6, 15]. However, in less than half of the studies (33%), HCWs reported needing psychological support to manage their mental health, suggesting thus HCWs might have wider needs than just individual. Psychological interventions that were found to be particularly effective on improving mental health symptoms such as anxiety, depression, and stress included ACT and CBT. Mindfulness-based exercises also appeared to be promising on improving depression symptoms. Our findings are in line to those of previous studies [6, 14, 24], suggesting that researchers and clinicians should use contextual approaches when intervening for the individual-based needs of HCWs to maximize and produce long-lasting effects.

Multi-level and multi-dimensional interventions should be preferred and based in accordance with reported guidelines [25]. Although there is a lack of studies implementing the socio-ecological framework when delivering interventions for improving the mental health of HCWs, some countries deliver socio-ecological interventions to non-HCWs populations (e.g., general population, families) for improving their mental health [74, 75]. For example, an ecological model of intervention for improving the mental health of individuals in Alberta [75], included educating individuals to manage their mental health (individual-level), group suicide intervention or mental health training (interpersonal-level), peer or social support groups (community-level) and suicide or mental health crisis lines (system-level). An additional example includes the combination of psychological interventions with medication use, that show promising results for managing mental health issues than using each of them alone [76, 77].

In addition, we found that although various apps and websites developed for HCWs resulted in improved mental health symptoms (e.g., PsyCovidApp, My Health too, Foundations, SH +, RECHARGE and RESTORE interventions) [56, 59–63], evidence is limited to a single study each. Thus, further evaluation of these digital-based interventions is required to strengthen their evidence base. Digital mental health applications are considered to be particularly effective for managing mental health problems such as depression, anxiety and schizophrenia, offering numerous benefits to the individuals (e.g., ease of habit, low effort expectancy) [78]. Additionally, the available interventions tend to be administered for a short duration, with the majority following HCWs for less than two months without concluding evidence on their long-term effectiveness. According to the American Psychological Association [79], on average, 15 to 20 sessions are required for 50% of patients to recover, suggesting thus the importance of administrating interventions for more than 2 months.

Importantly, HCWs reported that most of their needs were organizational such as flexible working hours, safe working conditions, paid rest periods, improvement of protective equipment, support by superiors and co-workers and clear communication of policies related to workload and sick leave. This suggests the important role that work environment plays in the mental health and well-being of HCWs and the crucial role of healthcare systems to provide adequate support to their employees [5, 6]. Workplace environment is also an important determinant of HCWs’ performance and productivity, with their satisfaction associated with high-quality care provision. For example, studies suggest that supervisor support, incentives, recognition and reward system could be used to improve HCWs’ experiences and their overall work satisfaction [80, 81]. Given the importance that workplace environment has to the mental health of HCWs [82, 83], improving only individual-based needs will result only in small and short-term improvements in HCWs’ well-being. During pandemic outbreaks, organizational support has been proven to be effective in protecting the mental health of HCWs by having a proper plan with supporting online platforms for HCWs to express and address their concerns and feelings [84]. If workplace needs of HCWs are not adequately supported, this may result in emotional exhaustion and thus possibly reduced quality care to their patients.

With respect to interpersonal-related challenges and needs, studies reported HCWs being unable to spend time and having to choose work over family, while expressing a need for support from their family, spouse, and friends. The need of support by family members was found to be a crucial factor for motivating HCWs’ to work during COVID-19, with reduced family support associated with HCWs’ reduced mental health and well-being [8, 14]. Due to the COVID-19 pandemic, HCWs had to stay away from their family to protect them and were forced to work long hours under pressure, leading then into reduced mental health and social isolation [5, 6]. Policies regarding the inclusion of family members in treatment could be promoted with provision of brief training or skills enhancement for family members [85].

Studies also reported that community-level challenges and needs included mostly stigma and discrimination from society while expressing a need for support from community members. Societal stigmatization of HCWs during COVID-19 is not surprising as previous research [2, 5, 9, 15] suggests that since the beginning of the COVID-19 pandemic, social prejudice and stigmatization was directed to HCWs as they were exposed to COVID-19 and community members feared that they would contract them COVID-19. Possible interventions at the community-based level might include educational campaigns on the measures required to control the spread of the virus. Acknowledging the significant contribution of HCWs by community members is crucial as providing support to HCWs during pandemics might enhance their resilience and possibly reduce their burnout. Although some efforts were deployed during the COVID-19 pandemic to recognize the contribution of HCWs [86, 87], more support is needed as it can improve the functionality of the healthcare system and the overall resilience of communities during health crises.

### Limitations

The results of this scoping review should be interpreted considering for its limitations. First, due to the rapid need for a review in this area, only three databases were searched, and a single reviewer extracted the data of the articles. However, the databases were chosen for their comprehensive coverage of health and psychological research, representing the main topic in a sufficient way. Furthermore, this review was limited to English language studies, thus, we might have missed some relevant studies especially from non-English speaking countries or journals. It should also be considered, that 51% of the included studies were conducted in non-English speaking countries (e.g., Pakistan, Bangladesh, Iran, Africa, India, Indonesia). In addition, some countries that were highly affected by the COVID-19 (e.g., Brazil, China) [88] were either underrepresented (e.g., only three studies included that were published in China) or no studies were identified (e.g., Brazil). No quality assessment was conducted as this study was a scoping review and therefore the evidence could be influenced by the studies’ methodological shortcomings.

### Implications for researchers and clinicians

This rapid scoping review is the first mapping of the challenges, needs and psychological interventions for HCWs caring for COVID-19 patients based on the socio-ecological models. Given that behavior change and mental health improvement entails an interaction of factors [72], multi-level and multi-dimensional interventions are needed addressing not only individual-based factors, but also the multiple socio-ecological levels with a variety of interventions (e.g., societal, workplace, family, group and individual). However, expecting any single intervention to focus on three or more ecological levels may be unrealistic, but given that HCWs are the first to be infected and that they are the key to a healthcare system’s ability to respond to pandemic outbreaks, it is crucial to implement interventions that incorporate at least the individual and organizational key members [89] while encouraging health care systems to adopt a stepped care approach to services [90, 91]. Adopting a stepped care approach to delivering of interventions might be particularly useful, with the degree of support that HCWs will receive being stepped up based on their needs or presence of psychological symptoms [90, 92]. Digitally delivered interventions hold promise for effectively improving mental health and well-being, and can be used when HCWs are socially isolated and for targeting the limited available time due to excessive workload [14].

Psychological interventions were only delivered in HCWs working in primarily urban areas (e.g., Ontario, Zurich, Istanbul, Okayama). Although interventions are suggested to be more impactful in urban areas [93, 94], it is important to examine their effectiveness for HCWs working in rural areas as rural residents were found to be less likely to adapt preventive COVID-19 measures than those in rural areas [95]. Future studies are suggested to utilize interventions based on the socio-ecological framework additionally in rural areas and examine whether regional differences exist on interventions’ efficacy [93].

Community psychology interventions might be also effective as individuals’ behavior is influenced by the interaction with their context [96, 97]. Specifically, including community members (e.g., co-workers) in treatment is essential. Community interventions that focus on community-level change rather than individual usually integrate social, cultural, economic, political, and environmental to achieve empowerment at individual and systemic levels. For example, an intervention approach for HCWs based on community psychology might include group prevention trainings with other HCWs to address fears and reduced mental health related to the COVID-19 pandemic, problems experienced at work, and social action strategies such as community education. By addressing the multiple levels of influence on HCWs’ needs, interventions are more likely to be effective and to possibly better cope with future pandemic situations.

## Data Availability

All materials related to the review are available by contacting the corresponding author.

## Appendix

### Search strategy

- Pubmed

(“healthcare”[Title/Abstract] AND (“professionals”[Title/Abstract] OR “providers”[Title/Abstract] OR “doctors”[Title/Abstract] OR “physicians”[Title/Abstract] OR “workers”[Title/Abstract] OR “nurses”[Title/Abstract]) AND (“covid-19”[Title/Abstract] OR “covid-19”[Title/Abstract] OR “SARS-COV-2”[Title/Abstract] OR “coronavirus”[Title/Abstract]) AND (“need”[Title/Abstract] OR “challenge”[Title/Abstract] OR “intervention”[Title/Abstract] OR “treatment”[Title/Abstract])) AND (2020:3000/12/12[pdat]).

b. Scopus

(TITLE-ABS-KEY (healthcare) AND TITLE-ABS-KEY (professionals) OR TITLE-ABS-KEY (providers) OR TITLE-ABS-KEY (doctors) OR TITLE-ABS-KEY (physicians) OR TITLE-ABS-KEY (workers) OR TITLE-ABS-KEY (nurses) AND TITLE-ABS-KEY (need) OR TITLE-ABS-KEY (challenge) OR TITLE-ABS-KEY (intervention) OR TITLE-ABS-KEY (treatment) AND TITLE-ABS-KEY (covid-19) OR TITLE-ABS-KEY (covid 19) OR TITLE-ABS-KEY (sars-cov-2) OR TITLE-ABS-KEY (coronavirus)) AND PUBYEAR > 2020.

c. CINAHL

TI-AB healthcare AND (TI-AB professionals OR TI-AB providers OR TI-AB doctors OR TI-AB physicians OR TI-AB workers OR TI-AB nurses) AND (TI-AB COVID-19 OR TI-AB covid 19 OR TI-AB SARS-COV-2 OR coronavirus) AND (TI-AB need OR TI-AB challenge OR TI-AB intervention OR TI-AB treatment) Date range: 2020-now (last 4 years).
